# Modified diatomite for soil remediation and its implications for heavy metal absorption in *Calendula officinalis*

**DOI:** 10.1186/s12870-024-05068-7

**Published:** 2024-05-03

**Authors:** Maryam Samani, Yogesh K. Ahlawat, Ahmad Golchin, Hossein Ali Alikhani, Ahmad Baybordi, Sadhna Mishra

**Affiliations:** 1 Soil Science Department, Faculty of Agriculture, University of Zanjan, Zanjan, Iran; 2 Centre of Research Impact and Outreach, Institute of Engineering and Technology, Chitkara University, Rajpura, Punjab 140401 India; 3 Centre of Research Impact and Outreach, Chitkara University, Baddi, Himachal Pradesh India; 4 Soil Science Department, Faculty of Agriculture, University of Tehran, Tehran, Iran; 5 Soil and Water Research Department, East Azerbaijan Agriculture and Natural Resources Research and Education Center, AREEO, Tabriz, Iran; 6 Faculty of Agricultural Sciences, GLA University, Mathura, Uttar Pradesh 281406 India; 7 Department of Agriculture sciences , Sharda University, Agra, Uttar Pradesh 282002 India

**Keywords:** Modified diatomite, Soil remediation, Heavy metals, Soil nutrients, *Calendula officinalis*

## Abstract

**Background:**

Among different adsorbents, natural and inorganic compounds such as diatomite are important and advantageous in terms of high efficiency and cost-effectiveness, and function in stabilizing heavy metals in the environment. *Calendula officinalis*, a plant known as a high accumulator of heavy metals, was cultivated in soil treated with varying concentrations of modified diatomite to demonstrate the efficiency of modified diatomite in stabilizating of heavy metals in soils,

**Results:**

The modification of diatomite aimed to enhance *Calendula officinalis* adsorptive properties, particularly towards heavy metals such as lead (Pb), Zinc (Zn), Chromium (Cr), Nickle (Ni), and Copper (Cu), common contaminants in industrial soils. The experimental design included both control and treated soil samples, with assessments at regular intervals. Modified diatomite significantly decreased the bioaccumulation of heavy metals in contaminated soils except Zn, evidenced by decreased DTPA extractable heavy metals in soil and also heavy metal concentrations in plant tissues. Using 10% modified diatomite decreased 91% Pb and Cu, 78% Cr, and 79% Ni concentration of plants compared to the control treatment. The highest concentration of Zn in plant tissue was observed in 2.5% modified diatomite treatment. Remarkably, the application of modified diatomite also appeared to improve the nutrient profile of the soil, leading to enhanced uptake of key nutrients like phosphorus (P) 1.18%, and potassium (K) 79.6% in shoots and 82.3% in roots in *Calendula officinalis*. Consequently, treated plants exhibited improved growth characteristics, including shoots and roots height of 16.98% and 12.8% respectively, and shoots fresh and dry weight of 48.5% and 50.2% respectively., compared to those in untreated, contaminated soil.

**Conclusion:**

The findings suggest promising implications for using such amendments in ecological restoration and sustainable agriculture, particularly in areas impacted by industrial pollution.

## Background

Due to the increasing soils pollution all over the world, the quest for innovative and effective methods to mitigate soil contamination has become increasingly crucial. Soil, a fundamental component of terrestrial ecosystems, often falls victim to heavy metal pollution, a consequence of industrial activities, agricultural practices, and improper waste management [1]. These contaminants pose a significant risk not only to environmental health but also to human well-being, as they can enter the food chain through crop uptake. Within this context, this study explores the use of modified diatomite as a soil remediant of heavy metal, focusing on its impact on the growth and development of *Calendula officinalis*, a widely cultivated medicinal and ornamental plant. Diatomite, a naturally occurring, siliceous sedimentary rock composed of fossilized remains of diatoms, has garnered attention for its potential in soil remediation due to its high porosity, large surface area, and unique adsorption properties. The modification of diatomite, aimed at enhancing its ability to bind heavy metals, could offer a sustainable and efficient solution for soil decontamination.

Marigold (*Calendula officinalis*), is chosen for this study due to its widespread use in pharmaceutical, cosmetic, and culinary industries, as well as its role as a bioindicator for soil health. The response of *Calendula officinalis* to the modified diatomite-treated soil provides valuable insights into the broader implications of using such soil amendments in agricultural and horticultural practices. Soil contamination with heavy metals is one of the most important environmental challenges in recent decades due to its negative effects on the environmental cycle and the physiological and metabolic activity of living organisms. Unlike organic pollutants, heavy metals are non-degradable and have a long half-life, which is why they hurt the soil ecosystem [2]. Absorption of high amounts of heavy metals by plants can cause human poisoning and cause many acute and chronic diseases. Excessive uptake of heavy metals by plants poses a risk of human poisoning and can lead to various acute and chronic illnesses. The elevated presence of heavy metals in the soil adversely affects crop growth. Ideally, soil tainted with heavy metals should undergo rehabilitation to eliminate pollution, allowing for its safe reuse [3]. However, restoring the soil’s original quality can be a costly endeavor. Therefore, selecting the most appropriate decontamination method depends not only on potential profitability and the future viability of the soil but also on an understanding of the challenges and obstacles involved. Various techniques exist for remediating soils contaminated with both mineral and organic pollutants. These methods encompass leaching and extraction processes utilizing different solvents, reverse osmosis, membrane separation, nanofiltration, ion exchange, deposition, and absorption by adsorbents [4]. All the below methods are based on one of the following two principles:

**(i)*Extraction process***: This process includes removing the pollutant from the soil, (ii)The process of immobilizing pollutants in the soil: In this process, immobilization includes the solidification or stabilization of the pollutant to prevent the spread of pollution to uncontaminated soils and underground water. In the process of chemical stabilization, various additives are used to reduce the mobility and availability of pollutants [5]. Using some of the mentioned methods to correct all polluted sources is a very difficult and costly task. Therefore, nowadays the request for using innovative and low-cost methods has increased. Among all the above methods, the absorption technique by absorbents is preferred over other methods. Because this method, in addition to being effective and fast, is a simple method from a technical point of view, a healthy and safe method from an environmental point of view, and a cost-effective method from the point of view of cost, and it has received more attention from water and soil science researchers [6]. The purpose of immobilization is to prevent the entry of toxic compounds into biological cycles by reducing the solubility or toxicity of these compounds. In this method, the contaminated soil is mixed with suitable compounds and modifiers, and then due to changes in pH, ion exchange absorption, and sedimentation processes, the dynamics, solubility, and toxicity of the pollutant are reduced. Among different adsorbents, natural and inorganic compounds such as diatomite are important and advantageous in terms of high efficiency and cost-effectiveness, and function in stabilizing heavy metals in the environment [1]. Diatomites are natural structures that have 80–90% porosity. Diatomites are colorless silica cement minerals that are formed from the microfossil shells of single-celled algae called diatoms and usually contain 86–94% silica [7]. Many scientists have used diatomite as an adsorbent to remove color from wastewater, and absorption of heavy metals. It also showed that the functional group – SH- SiO_2_ can effectively remove lead, nickel, and mercury heavy metals from the environment [8]. Many researchers believe that the specific surface area and the size of the pores of silica-based materials are effective in their ability to absorb metals. Natural diatomite has a high specific surface, which indicates their high adsorption and chemical absorption capacity [9, 10]. The adsorption capacity of diatomite increases through the modification of surface layers and improves the adsorption efficiency of diatomite [9]. Diatomite is utilized as a cost-effective adsorbent for removing metals from wastewater [11]. Its efficiency in metal ion adsorption is due to its distinctive physical and chemical characteristics, including a highly porous structure, low thermal conductivity, effective sorption capacity, inertness, low density, and a large surface area [12]. Furthermore, diatomite successfully immobilized heavy metals in contaminated acidic soils. The bioavailable index (BI) values of metals like Zn, Cu, Cr, and Ni were reduced in acidic soils contaminated with heavy metals through the amendment with sepiolite, zeolite, and diatomite [13]. Additionally, modified diatomite could be effective in immobilizing certain metals in polluted soils [14]. Marigold, *Calendula officinalis*, belongs to the Asteraceae family and originates from the Mediterranean region. It is one of the high-accumulating plants that has shown a high ability to absorb heavy metals and has been used in many types of research for phytoremediation [15, 16]. Therefore, it can be used as an indicator plant to evaluate the ability of modifiers to stabilize and immobilize heavy metals in the soil.

## Materials and methods

To assess the effectiveness of altered diatomite in diminishing the presence of heavy metals such as lead, zinc, copper, chromium, and nickel in soil, as well as its impact on the growth characteristics and concentration of heavy metals, phosphorus, and potassium in marigold plants, a greenhouse experiment was conducted using a completely randomized design. A composite soil sample was gathered from a depth of 0–15 cm within an urban park in Tehran, Iran. This sample was air-dried, sieved through a 2 mm mesh, subjected to analysis for physicochemical properties, and utilized in the investigation.

### Soil analysis

Soil texture was determined by the hydrometer method [17]. The pH of saturated paste (pHs) and the electrical conductivity of saturated extract (ECe) were measured using a pH meter model 3510 JENWAY and an EC meter model Cond 80 Pro respectively [18, 19]. soil organic carbon (OC%) was determined by the wet combustion method using potassium dichromate and sulfuric acid [20]. Cation exchange capacity (CEC) was calculated using the sodium acetate method [21]. The available fractions of heavy metals in the soil were extracted by DTPA [22], and their concentrations were determined using ICP-MS. the concentrations of total heavy metals in the soil, after soil digestion by nitric acid and hydrochloric acid were measured by ICP-MS [23],.

### Greenhouse study

To evaluate the effect of modified diatomite on heavy metal contaminated soil a greenhouse experiment was carried out using compeletly randomized design with three replications. The treatments involved blending modified diatomite : 0, 2.5, 5, 7.5, and 10% w/w. Both treated and untreated soil samples were then incubated for two months at field capacity moisture. After this period, marigolds were planted in each pot. During the 75-day growth cycle, the plants were irrigated with distilled water to maintain soil moisture at field capacity. This was achieved by daily weighing of the pots. After growing period, the shoots and roots parts of the plants were collected, the height of the plants and the wet weight of both shoots and roots were recorded. The plant samples were dried in an oven for 72 h at 60℃. Then weighed and sieved. Then plant material was digested using concentrated nitric acid and 30% hydrogen peroxide at 120 °C [24]. The extracts were analyzed for heavy metal concentrations using ICP-MS. Additionally, phosphorus levels were measured using a CE 292 Digital UV-Visible spectrophotometer, and potassium levels were determined with a Jenway PFP7 flame photometer.

### Soil amendment

Natural diatomite was prepared from Madan Kavan company provide natural diatomite from mine in Birjand, Khorasan province, Iran.

### Purification of natural diatomite by chemical method

A chemical method was used to remove impurities from the natural diatomite. Samples of natural diatomite were added to the 5 M hydrochloric acid solution 1:20 solid-to-liquid mixture, then the mixture was heated to reach boiling point (95° C). The mixture was stirred vigorously for 24 h. Then the supernatant was discarded and the remaining solids were washed several times with distilled water and dried at 105℃ for 2 h [25]. The particle size of purified diatomite was reduced to nano-scale by grinding with a ball mill for 10 h at 250 rpm [26].

### **Statistical analysis**

The data were analyzed using the SPSS 21.0 statistical software package and Excel 2016 for Windows. The means of three replicates were subjected to one-way ANOVA using the Duncan test at the 0.05 confidence level. The experiment was conducted based on a completely randomized design (CRD). The treatments included five levels of modified diatomite at levels (0, 2.5, 5, 7.5, 10% w/w) in three replicates. The completely random design was chosen because the base of the samples was equal and the only variable factor was the treatment level of modified diatomite. The number of treatments and the number of repetitions were balanced CRB. The mean comparison was done through Duncan’s test at 5% confidence level.

## Results

### Soil characteristics

Some physicochemical properties of the soil used in this study are summarized in Table 1. According to findings, the soil had a Silty Loam texture, relatively neutral pH, and low EC and OC content.

**Table 1:** Some physical and chemical properties of the soil used in this study

Texture	Silty loam
Sand (%)	38
Silt (%)	51
Clay (%)	11
pH	7.47
EC (dS m^− 1^)	0.45
CEC (Cmol_c_ kg^− 1^)	14.8
OC (%)	0.6
SSA(m^2^g^− 1^)	19.63
Available Pb (mg kg ^− 1^)	7.54
Available Zn (mg kg ^− 1^)	27.12
Available Cu (mg kg ^− 1^)	7.75
Total Pb (mg kg ^− 1^)	59.82
Total Zn (mg kg ^− 1^)	200.95
Total Cu (mg kg ^− 1^)	61.5

### Characteristics of modified diatomite used in this study

#### The results of chemical analysis of modified diatomite by XRF

The results of the chemical analysis of modified diatomite by XRF are shown in Table 2. The reduction in the amount of aluminum oxide by acid washing was 50.86% as compared to natural diatomite and reached 5.39%. Similarly, iron oxide reached 0.73% in the modified diatomite.

**Table 2:** Chemical compounds in natural and modified diatomite based on XRF analysis

	K_2_O	CaO	Na_2_O	Fe_2_O_3_	Al_2_O_3_	SiO_2_	MgO
Modified diatomite	1	0.54	0.82	0.73	5.39	83.19	0.37
Natural diatomite	1.27	3.43	1.62	3.29	10.97	59.43	4.19

#### XRD Results of modified diatomite

**Fig. 1:**
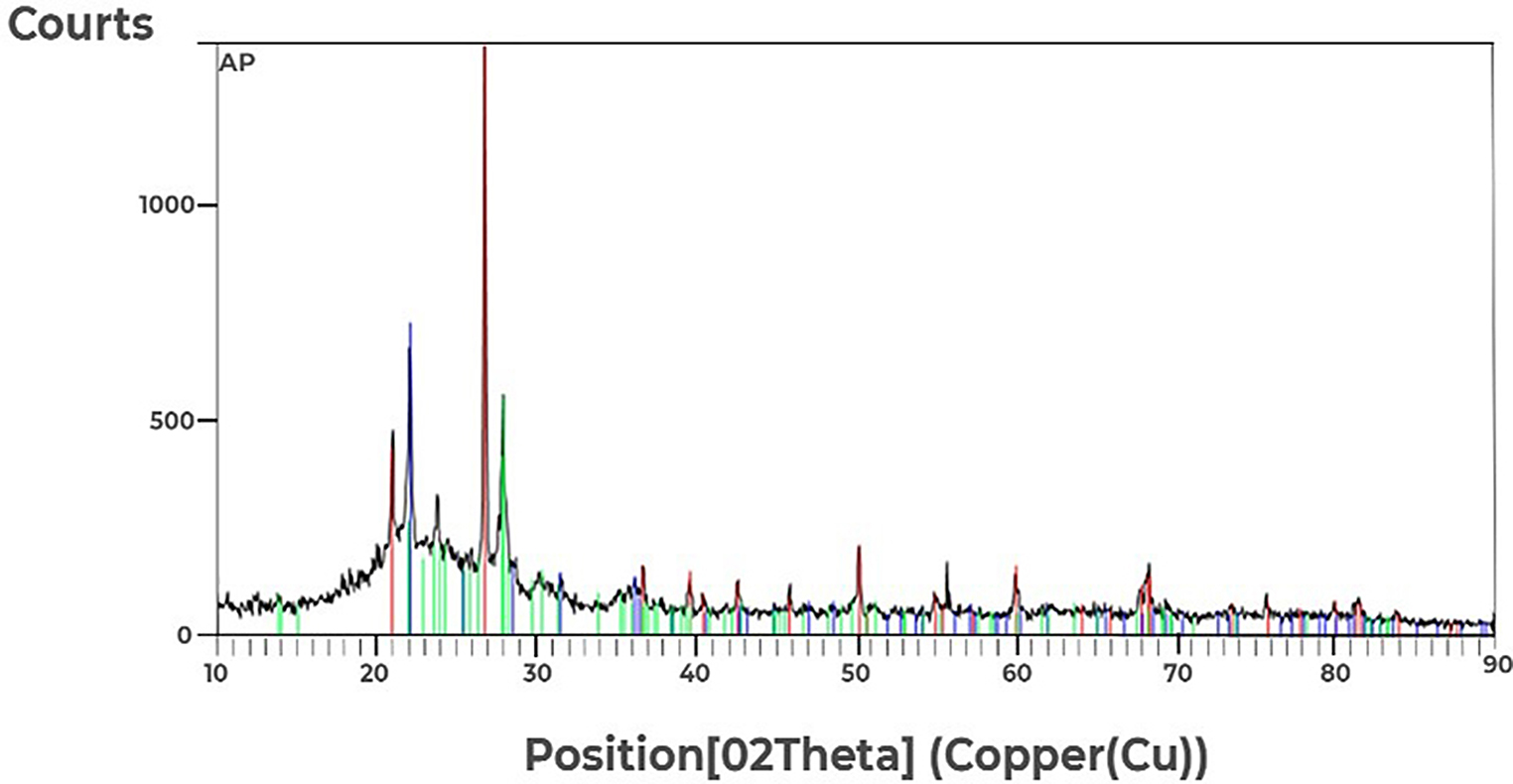
XRD pattern of modified diatomite demonstrated a crystalline structure of SiO_2_, also albite mineral. SiO_2_ is not dissolved in the acid-washing process. Albite is also an aluminosilicate mineral that will not dissolve in the acid-washing process will remain acidic solutions and its structure will remain unchanged

The modified diatomite sample, which was analyzed by XRD after the purification process (Fig. 1), has a crystalline structure of SiO_2_ (quartz) with sharp peaks at 20.9, 26.7, and 36.6. 50.2 and 60.1 of 2θ. Maximum peaks are also observed in 21.9, 31.4, 36.1, and 47 of 2θ, related to albite mineral ((Na, Ca) Al (Si, Al) _3_). The reason for the presence of SiO2 structure in the sample after the purification process can be stated that this structure is not dissolved in the acid-washing process Albite is also an aluminosilicate mineral that will not dissolve in acidic solutions and its structure will remain unchanged.

#### The SEM results of natural and modified diatomite

**Fig. 2:**
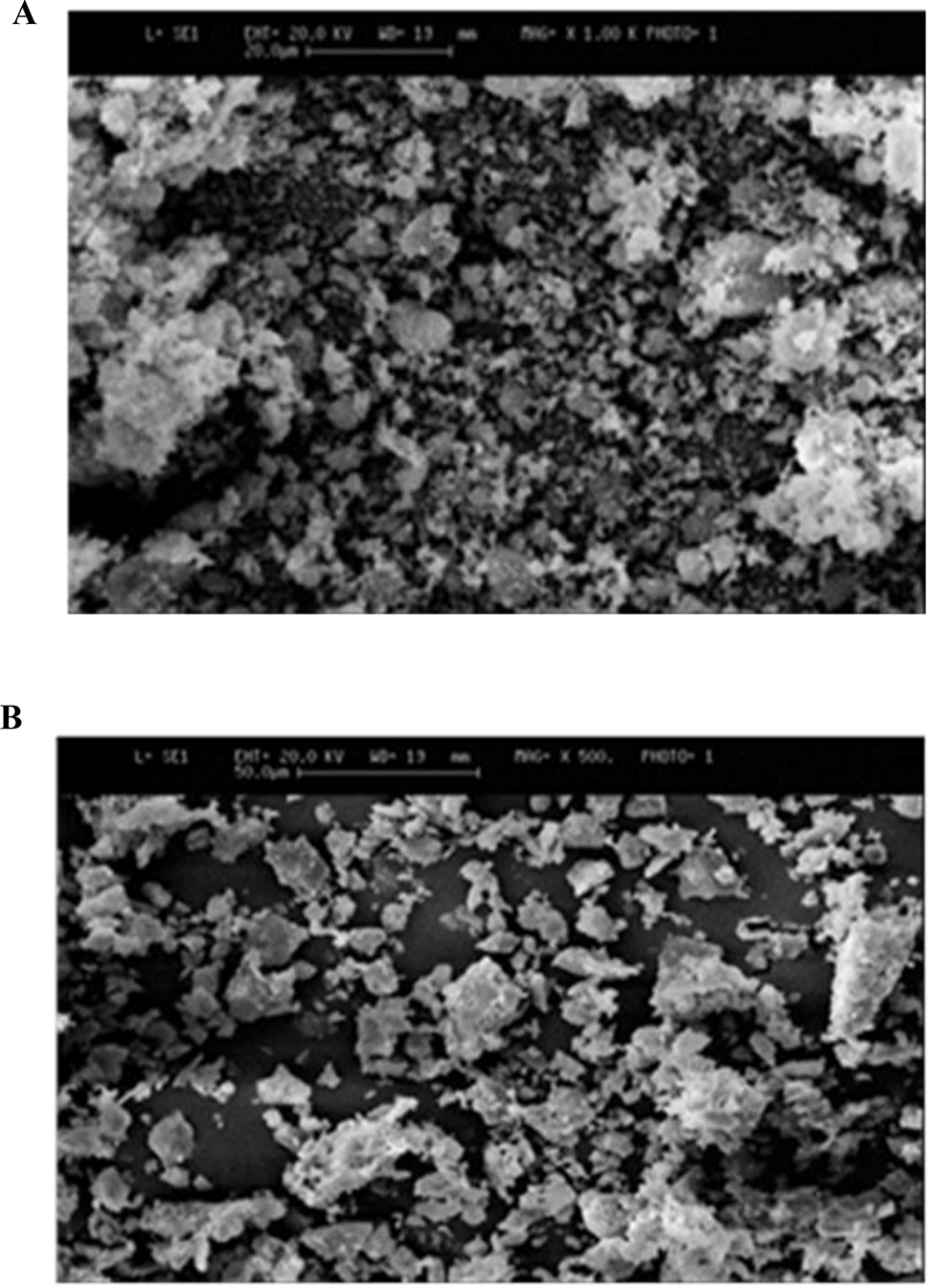
The surface morphology of particles of natural diatomite(**A**) and modified diatomite (**B**) by SEM. natural diatomite had more impurities in structure

The surface morphology of particles of natural (A) and modified diatomite (B) by SEM is demonstrated in Fig. 2, natural diatomite had more impurities in structure. In Fig. 2A, numerous small white impurities are visible in the photo, whereas in Fig. 2B, these minute white entities are absent, revealing a predominantly black background. This indicates the successful removal of impurities through acid and heat treatment. Several studies on diatomite acid treatment, demonstrated similar outcomes in SEM images [12, 27]. The diatomite was predominant with the Pennate and Centric exoskeletons. Diatomite biopolymer is composed of the skeletal deposition of single-celled algae called diatoms with more than 10,000 species. Each species has distinct shapes and sizes between less than 5 microns and more than 100 microns. Many diatomite species are restricted to areas with specific pH, salinity, and nutrients. In a study on the removal of heavy metals from contaminated water, Pennate and Centric diatoms from Jordanian sources were used [28]. In another study on the stabilization of lead metal in soil the diatomite particles prepared from Khorasan mine had oval and round shapes [29].

n modified diatomite and acid wash process decreased the structural impurities.

#### Modified diatomite affectson the concentrations of DTPA extractable metals and soil pH

**Fig. 3:**
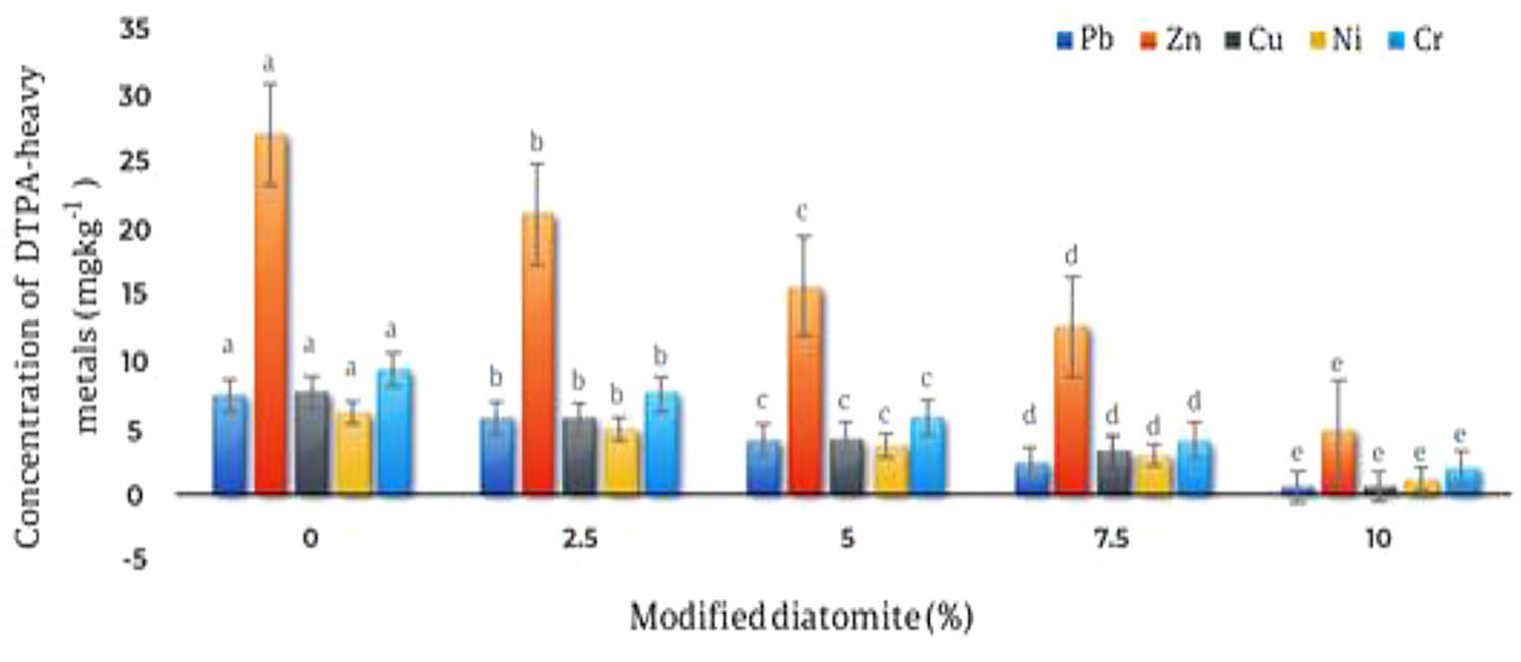
Effect of different levels of modified diatomite on the concentration of DTPA extractable metals. The highest reduction in the concentrations of DTPA extractable metals was acquired when the modified diatomite was applied at the rate of 10% w/w

The results showed that modified diatomite significantly (*p* ≤ 0.01) decreased the concentrations of DTPA extractable Pb, Zn, Cu, Cr and Ni, compared to unamended treatments (Fig. 3). the highest reduction in the concentrations of DTPA extractable metals was 10% w/w modified diatomite. The application of 10% modified diatomite decreased the concentrations of DTPA extractable Pb, Cu, Ni, Cr and Zn by 91.1%, 91%, 79.9%, 78.3% and 82.04% respectively (Table 3).

**Table 3:** The effects of different levels of modified diatomite (MD) on the DTPA extractable Pb, Zn, Cu, Ni and Cr of the tested soil

Extractable heavy metal DTPA (mg kg^− 1^)					
MD content (% w/w)	Cu	Zn	Pb	Cr	Ni
0	^a^ 7.75	27.12^a^	^a^ 7.54	9.42^a^	6.24^a^
2.5	^b^ 5.73	^b^ 21.15	^b^ 5.8	7.63^b^	5^b^
5	^c^ 4.29	^c^ 15.72	^c^ 4.14	5.84^c^	3.75^c^
7.5	^d^ 3.3	^d^ 12.74	^d^ 2.41	4.7^d^	3.02^d^
10	^e^ 0.69	^e^ 4.87	^e^ 0.67	2.04^e^	1.25 ^e^

Averages that have at least one letter in common are not statistically significant.

**Fig. 4:**
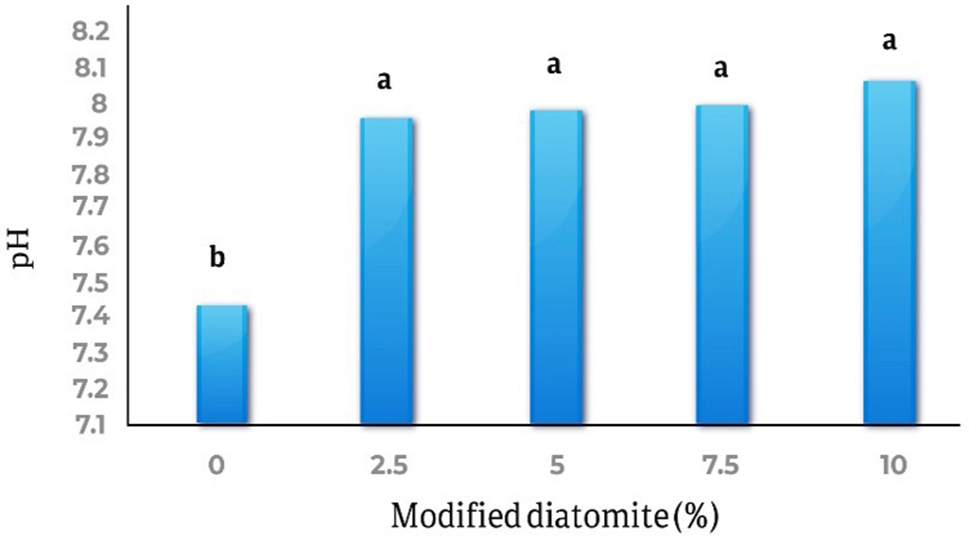
The changes in soil pH after the addition of modified diatomite

The results demonstrated that the use of modified diatomite significantly increased soil pH, compared to unamended control (Fig. 4). The pH of soils treated with 10% modified diatomite increased from 7.40 to 8.06.

#### The effect of different amounts of modified diatomite on *Calendula officinalis*

**Fig. 5:**
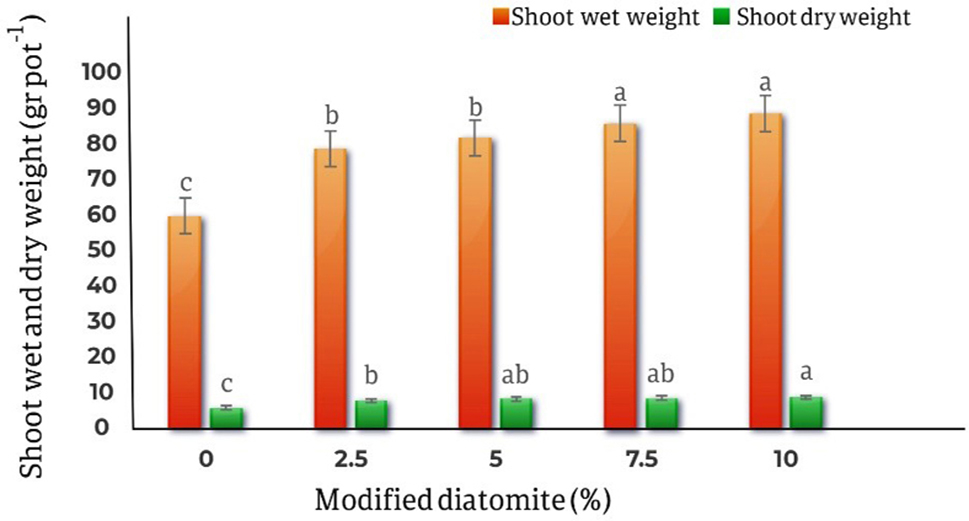
Effect of different levels of modified diatomite on the wet and dry weight of *Calendula officinalis* shoots

The results of different levels of modified diatomite on shoots fresh weight showed that the lowest shoots fresh weight belong to control treatment (59.5 gr/pot ), while the highest shoots fresh weight observed in the treatment 10% diatomite (88.36 gr/pot ), which has increased by 48.5% compared to the control treatment. Similarly showed that the lowest dry weight of the shoots is related to the control treatment and is 5.81 gr/ pot and the highest amount of shoots dry weight was 8.73 gr/ pot in the treatment of 10% diatomite, which was a 50.2% increase compared to the control (Fig. 5).

**Fig. 6:**
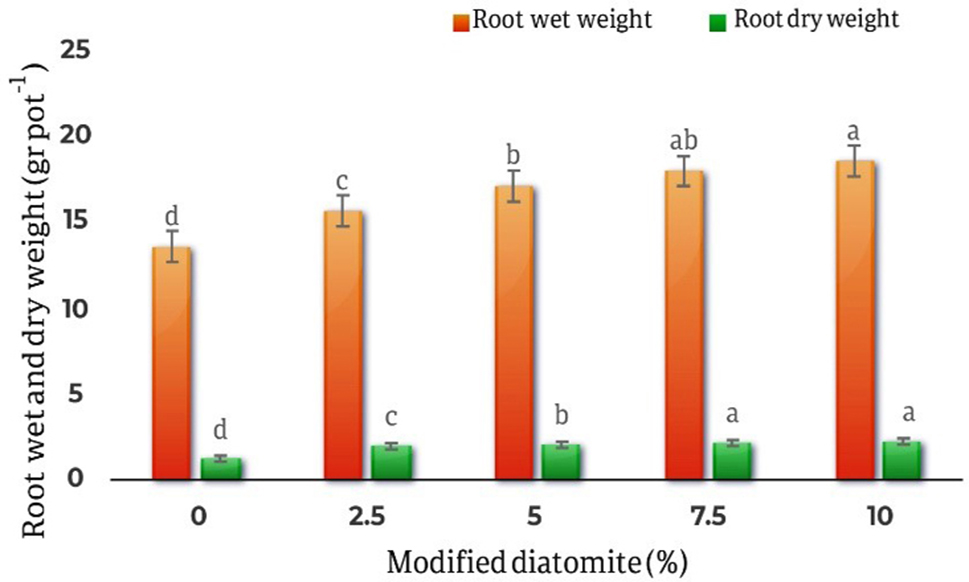
Comparison of the average effect of different levels of diatomite on wet and dry weight of *Calendula officinalis* roots. The highest amount of roots fresh weight is in the treatment of 10% diatomite which has increased by 36.7% compared to the control treatment. The highest amount of dry weight of the shoots was in the treatment of 10% diatomite, which was a 77.6% increase compared to the control.The comparison results of the averages of the effects of different diatomite levels on the length of *Calendula officinalis* shoots showed that the lowest length of the shoots was related to the control treatment with23.21 cm, and the highest length of the shoots was related to the treatment 10% w/w diatomite and equal to 27.96 cm. of the effects of different levels of diatomite on the roots length of the plant showed that the lowest roots length was related to the control treatments and equal to 11.23 cm, and the highest roots length was related to the treatment of 10% of diatomite and equal to 12.88 cm (Fig. 7)

The results of the averages comparing the effect of different levels of modified diatomite on the fresh and dry weight of the roots of *Calendula officinalis* showed that the lowest fresh weight of the roots is related to the control treatment and is equal to 13.55 gr/ pot, and the highest fresh weight of the roots is related to the 10% diatomite treatment, and it was equal to 18.53 gr/ pot, which was 36.7% higher than the control. Also, the comparison results of the average effect of diatomite on the dry weight of the roots showed that the lowest dry weight of the roots was related to the control treatment and equal to 1.25 gr/ pot, and the highest dry weight of the roots was related to the treatment of 10% by weight of diatomite. It was 2.22 gr/ pot with a 77.6% increase compared to the control (Fig. 6).

**Fig. 7:**
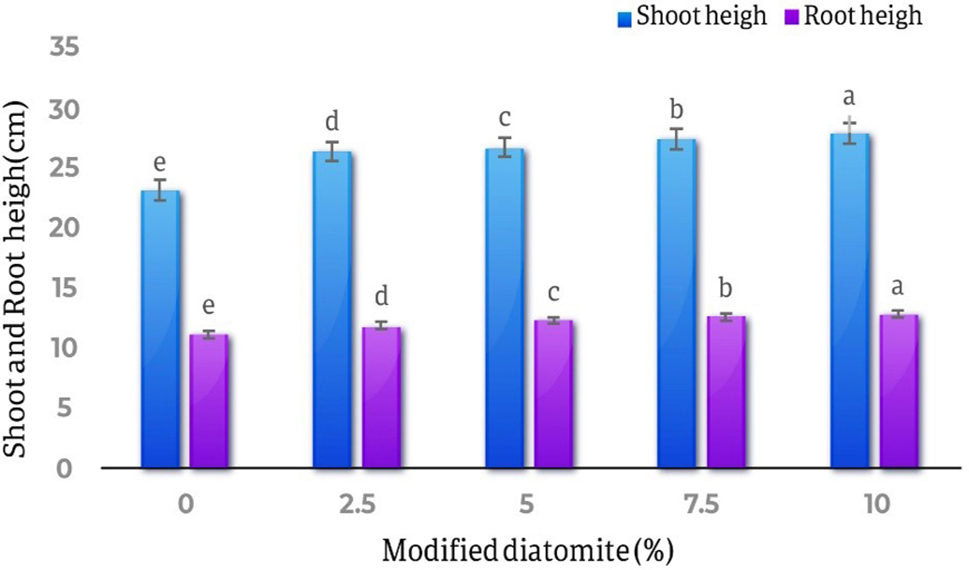
Comparison of the average effect of different levels of diatomite on the length of shoots and roots of *Calendula officinalis*. The highest length of roots and shoots was related to 10% diatomite and showed 12.8% and 16.98% increase respectively

### The effect of different treatments of diatomite on phosphorus and potassium absorption by *Calendula officinalis*

Different levels of diatomite affect the amount of phosphorus and potassium in the shoots and roots at the level of 1% (*p* < 0.01) and the effect of different levels of diatomite on the phosphorus of the *Calendula officinalis* roots was not statistically significant.

**Fig. 8:**
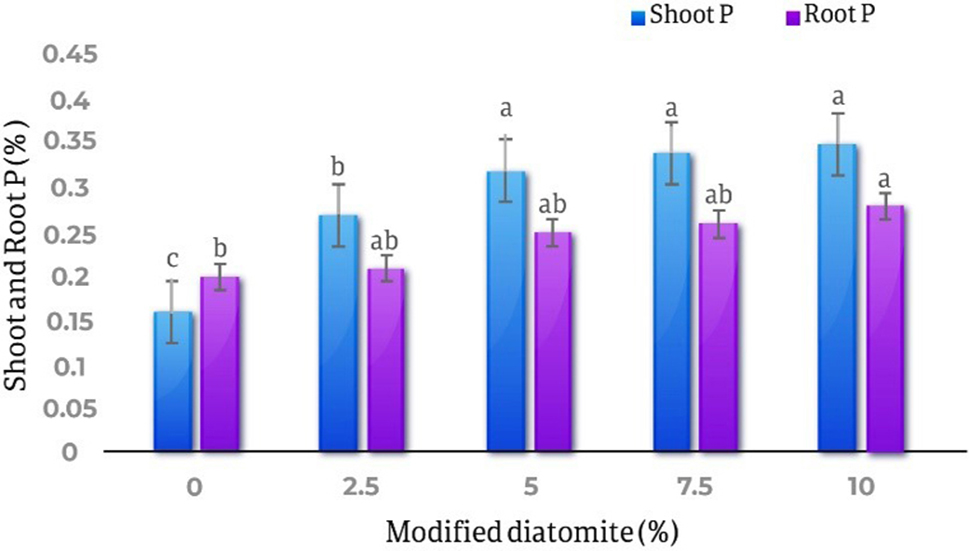
Comparison of the average effect of different levels of diatomite on the amount of phosphorus in the shoots and roots of *Calendula officinalis*. By increasing the amount of absorbent, the amount of phosphorus in the shoots and roots increases 1.18% and 40% respectively

The results of comparing the averages of the effect of different diatomite levels on the amount of phosphorus absorption in the shoots and roots of *Calendula officinalis* showed that with the increase in the amount of absorbent, the amount of phosphorus in the shoots and roots increased. It was found that the lowest amount of phosphorus in the shoots was in the control treatment and equal to 0.16% and the highest amount of phosphorus in the shoots was in the treatment of 10% diatomite and equal to 0.35%, which is 1.18% increased compared to the control treatment. The result of comparing the averages of the effect of diatomite on the amount of phosphorus in the roots showed that the lowest amount of phosphorus in the control treatment was 0.21% and the highest amount of phosphorus in the roots was in the treatment of 10% w/w with an increase of 40% compared to the control (Fig. 8).

**Fig. 9:**
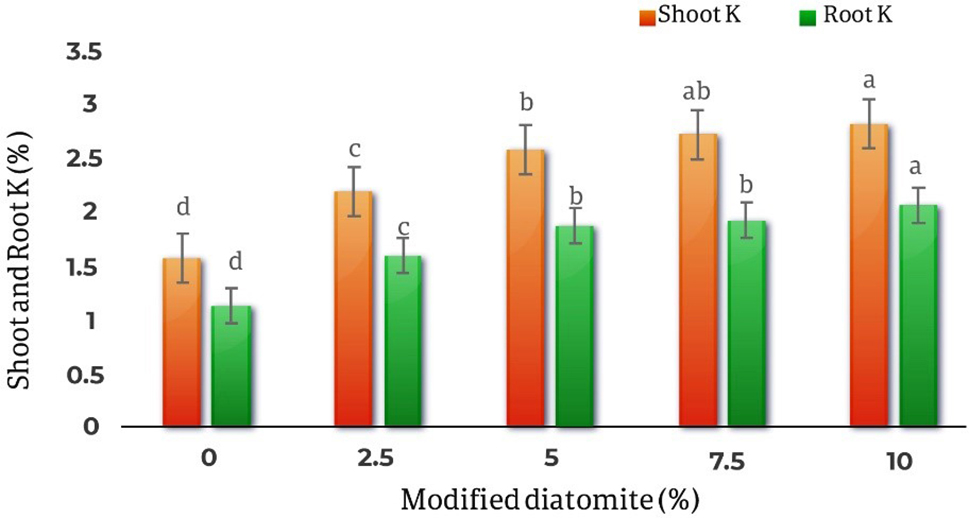
Comparison of the average effect of different levels of diatomite on the amount of potassium in the shoots and roots of *Calendula officinalis.* By increasing the amount of absorbent, the amount of Potassium in the shoots and roots increases by 79.61% and 82.3% respectively

The results of the comparison of the average effects of different diatomite levels on the amount of potassium absorption in the shoots and roots of the plant showed that with the increase in the amount of absorbent, the amount of potassium in the shoots and roots increased. It was found that the lowest amount of potassium in the shoots observed in the control treatment and equal to 1.57% and the highest amount of potassium in the shoots observed in the treatment of 10% (w/w) and was 79.61% more than the control treatment. Alsothe effect of diatomite on the amount of roots potassium showed that the lowest amount of roots potassium was in the control treatment 1.135% and the highest amount of roots potassium was in the 10% with an increase of 82.3% compared to the control and was equal to 2.06% (Fig. 9).

### The effect of modified diatomite on the absorption of heavy metals by the shoots and roots of the *Calendula officinalis*

The results of the variance analysis of the data showed that different levels of diatomite had a significant effect on the amount of metal absorption in the roots and shoots of the plant at a level of 1% (*p* < 0.01).

### Lead in roots and shoots

**Fig. 10:**
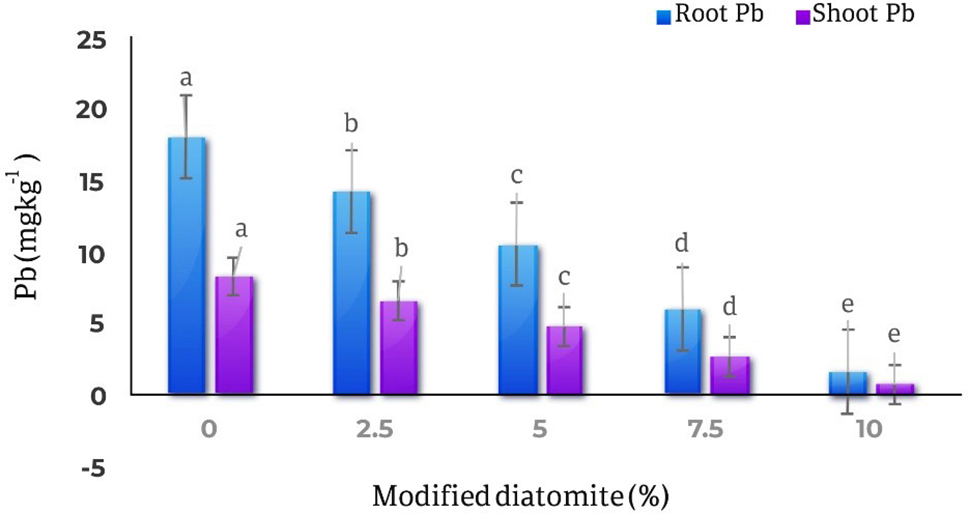
Comparison of the average effect of different levels of diatomite on the amount of lead in the roots and shoots parts of *Calendula officinalis*. By the 10% diatomite Pb concentration in both roots and shoots decreased about 91% compare to control

The results of comparing the averages of the effect of different levels of modified diatomite on the amount of lead in the roots and shootsof the *Calendula officinalis* showed that with the increasing in the amount of diatomite, the amount of lead in the roots and shoots parts of the plant decreased significantly. The highest amount of lead in roots was observed in the control treatment (18.73 mg kg^− 1^) and the lowest amount of lead in roots (1.68 mg kg^− 1^) was observed in the treatment of 10% w/w of modified diatomite, which is 0.3% compared to the control treatment. It decreased by 91%. Also, the highest amount of lead in the shoots part of the plant was 8.42 mg kg^− 1^in the control treatment and the lowest amount of lead in the shoots part was 0.75 mg kg^− 1^in the treatment of 10% w/w of diatomite with 91% reduction compared to the control (Fig. 10).

### Zinc in roots and shoots

**Fig. 11:**
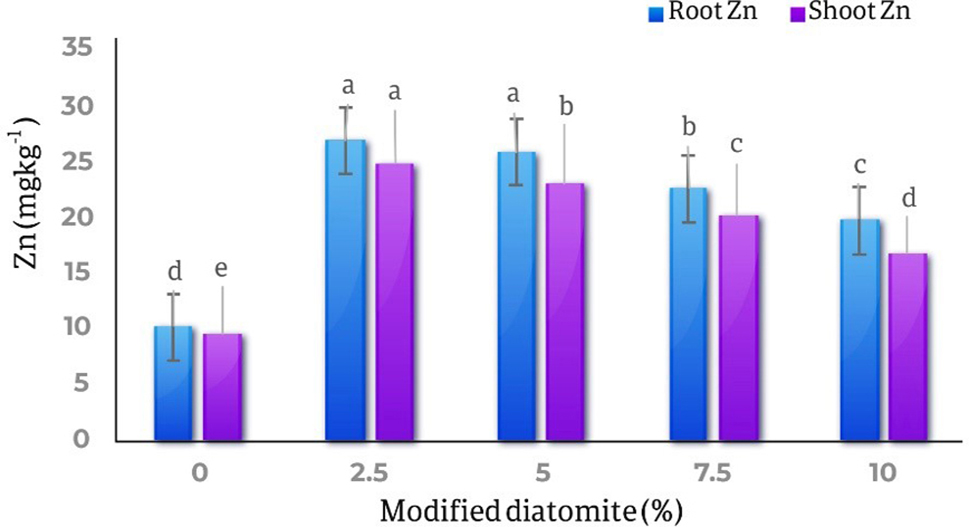
Comparison of the average effect of different levels of diatomite on the amount of zinc in the roots and shoots parts of *Calendula officinalis*. the highest amount of zinc in roots and shoots was at the treatment 2.5% of diatomite and increased by 1.6 times compared to the control treatment

The results of comparing the averages of the effect of different levels of diatomite on the amount of zinc in the roots and shoots parts of the marigold plant showed with increase in the amount of diatomite, the amount of zinc in the roots and shoots parts of the plant increased significantly. The highest amount of zinc in the roots was observed in the treatment of 2.5 and 5% w/w (26.72 mg kg^− 1^) and the lowest amount of zinc in the roots (10.04 mg kg^− 1^) was observed in the control treatment, the amount of zinc in the treatment 2.5% of diatomite increased by 1.6 times compared to the control treatment. Also, the highest amount of zinc in the shoots part of the plant was in the treatment of 2.5% of diatomite and equal to 24.64 mg kg^− 1^, and the lowest amount of zinc in the shoots part was in the control treatment and equal to 9.4 mg kg^− 1^. that the amount of zinc in the shoots in the treatment of 2.5% of diatomite increased by 1.6 times compared to the control. By increasing the amount of diatomite (5, 7.5, and 10% w/w), the amount of zinc in the shoots parts decreased compared to the 2.5% treatment (Fig. 11).

### Copper in roots and shoots

**Fig. 12:**
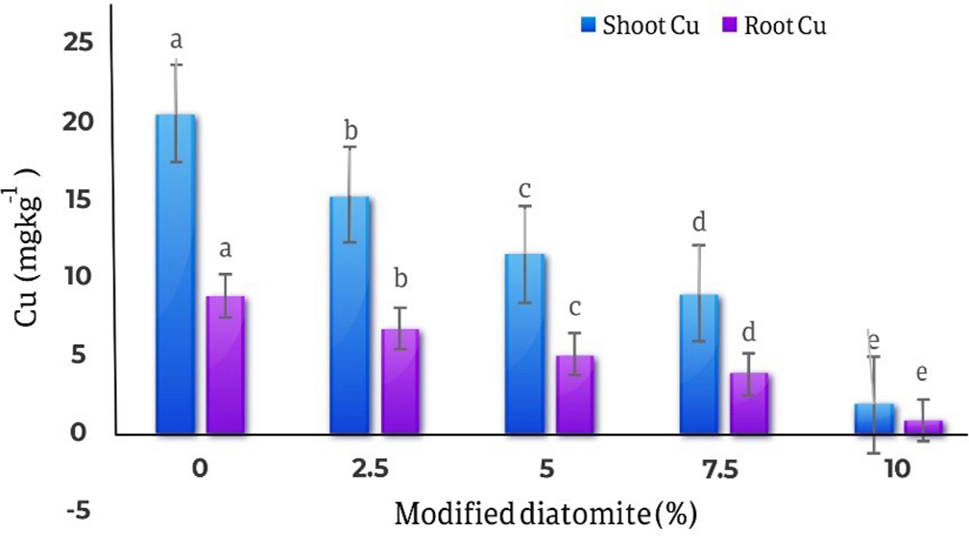
Comparison of the average effect of different levels of diatomite on the amount of copper in the roots and shoots parts of *Calendula officinalis*. By 10% modified diatomite, the concentration of Cu in roots and shoots decreased 91% compared to the control treatment

The results of comparing the averages of the effect of different levels of diatomite on the amount of copper in the roots and shoots parts of the marigold plant showed with the increase in the amount of diatomite, the amount of copper in the roots and shoots parts of the plant decreased significantly. The highest amount of copper in roots was observed in the control treatment (20.43 mg kg^− 1^) and the lowest amount of roots copper (1.83 mg kg^− 1^) was observed in the treatment of 10% of diatomite, with 91% decrease compared to the control treatment. Also, the highest amount of copper in the shoots part of the plant was 8.79 mg kg^− 1^in the control treatment, and the lowest amount of copper in the shoots part was 0.79 mg kg^− 1^in the treatment of 10% diatomite which is 91% decrease compared to the control treatment (Fig. 12).

### Chromium in roots and shoots

**Fig. 13:**
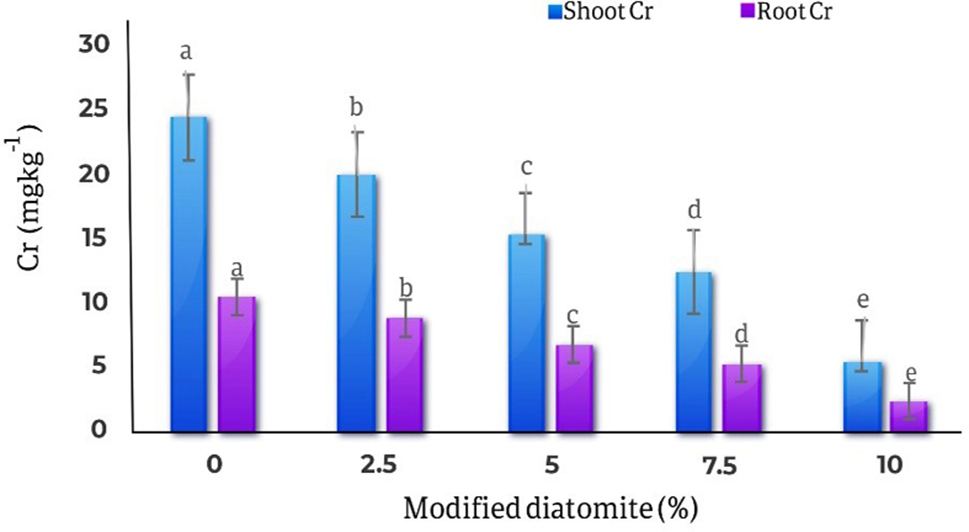
Comparison of the average effect of different levels of diatomite on the amount of chromium in the roots and shoots parts of *Calendula officinalis*. By 10% modified diatomite, Cr concentration in both roots and shoots decreased 78% compared to the control treatment

The results of comparing the average effects of different levels of diatomite on the amount of chromium in the roots and shoots parts of the marigold plant showed with the increase in the amount of diatomite, the amount of chromium in the roots and shoots parts of the plant decreased significantly. The highest amount of roots chromium was observed in the control treatment (24.38 mg kg^− 1^) and the lowest amount of roots chromium (5.33 mg kg^− 1^) was observed in 10% of diatomite treatment, and decreased by 78%. Also, the highest amount of chromium in the shoots part of the plant was 10.4 mg kg^− 1^ in the control treatment, and the lowest amount of chromium in the shoots part was 2.27 mg kg^− 1^ in the treatment of 10% w/w diatomite, which is 78% decreased compared to the control. (Fig. 13).

### Nickel in roots and shoots

**Fig. 14:**
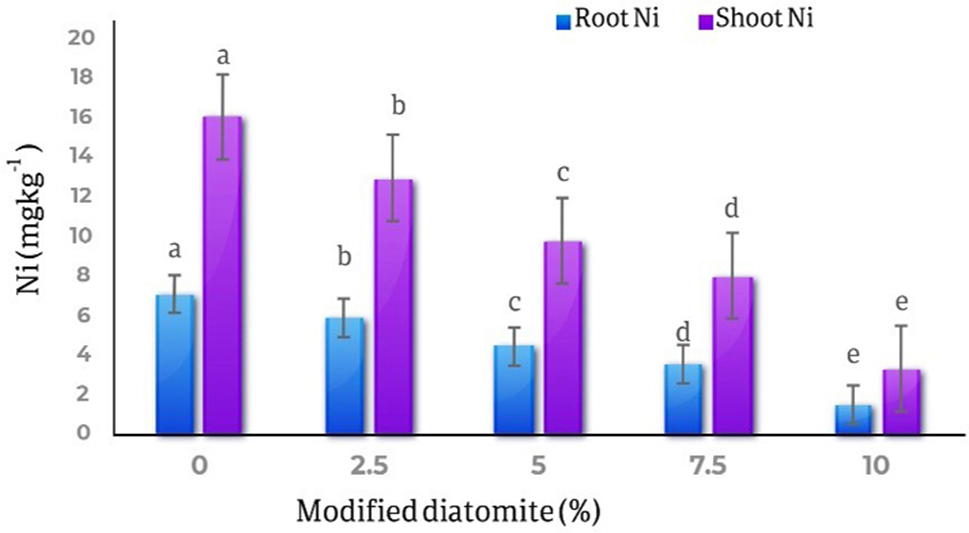
Comparison of the average effect of different levels of diatomite on the amount of nickel in the roots and shoots parts of *Calendula officinalis.* The lower concentration of Ni observed in the control treatment and by 10% modified diatomite, decreased by 79% in both roots and shoots

The comparison results of the average effects of different levels of diatomite on the amount of nickel in the roots and shoots parts of the marigold plant showed that with the increase in the amount of diatomite, the amount of nickel in the roots and shoots parts of the plant decreased significantly. The highest amount of nickel in roots was observed in the control treatment (7.02 mg kg^− 1^) and the lowest amount of roots nickel (1.42 mg kg^− 1^) was observed in the treatment of 10% of diatomite, which is 79.7% decreased compared to the control treatment. Also, the highest amount of nickel in the shoots part of the plant was 15.98 mg kg^− 1^ in the control treatment, and the lowest amount of nickel in the shoots part was 3.23 mg kg^− 1^ in the treatment of 10% diatomite, which is 79% decreased compare to the control (Fig. 14).

## Discussion

In this research, natural diatomite modified by HCl chemical method. the chemical modification of the natural diatomite by hydrochloric acid and heat reduced its impurities. According to the type of diatomite, acid with a certain concentration was used to activate the diatomite and remove impurities. In diatomite purification process, two different processing routes were examined: (i) leaching of the diatomaceous earth directly in cold acidic solutions of HCl; (ii) leaching of the diatomaceous earth directly by using hot 1 and 2 N HCl. It was found that all of the undesirable elements in the composition of the diatomaceous earth were dissolved when treated at about 100 °C with acidic solutions prepared using 1 and 2 N HCl for various leaching times. Under several cold acid concentrations, varying from 1 N to 5 N HCl, however, only a small amount of silica is dissolved, while significantly reducing the contribution of undesirable aluminium, calcium, magnesium, iron and alkaline element. The purification steps to remove the undesired organic and inorganic impurities involve generally the treatment of the diatomite in hot-acid solutions (e.g. HCl) at 75℃., The HCl concentrations and the acid leaching treatment times are strongly dependent on the impurity content of the source of rawdiatomite. Acid treatment enriches the raw diatomite by further removing the impurities from the surface pores, unclog the pores and makes new pores on the surface [29].

Results of this research showed that modified diatomite decreased the concentrations of DTPA extractable Pb, Zn, Cu, Cr and Ni, compared to unamended treatments. It seems that the application of 10% modified diatomite provided sufficient surfaces for the adsorption of heavy metals thus decreasing their concentration in the soil solution. The surfaces of diatomite have hydroxyl active groups that have high adsorption capacity and are in the forms of free silanol (Si-OH) groups, free silanol diol groups (Si -(OH)_2_) and atomic bridges with oxygen ions (Si-O-Si) in surface [30]. Silanol groups on the diatomite’s silica surface react easily with various agents. The adsorption capacity of the diatomite depends on the charge and electronegativity of the metals; the metal cations in the solution form a chemical bond with the siloxane oxygen attached to the surface of the diatomite. Both silanol and siloxane groups in the diatomite surface play a very important role in the adsorption capacity of metals [31]. The difference in the amounts of Pb, Cu, and Cd adsorbed may be due to the acid-base theory. Lead belongs to hard acids and tends to complex with hydroxy (hard base) groups on the surface of diatomite more than Copper and Cd. Thus, lead is more prone to immobilization than Copper and Cd. Reducing the hydrated radius and energy, and increasing the electronegativity increases the tendency to ion-specific adsorption. The reason for the higher adsorption of lead and copper ions than zinc is likely due to the lower hydrated radius of the Pb (0.401 nm) and Cu (0.419 nm) compared to the Zn (0.43 nm), and their higher electronegativity [12].

In this research, modified diatomite had a significant effect on soil pH, the soil pH is one of the important factors in controlling the balance between metal solutions in soil [32]. It is also reported that increasing pH reduces metals in mobile fractions and reduces the bioavailability of heavy metals in soil [25]. Other researchers have also reported that applying adsorbents that increase soil pH, reduces mobility and bioavailability of heavy metals, including Cd, Pb, Cu, and Zn [33]. The zeta potential of diatomite samples was different at various pHs and diatomite levels, and with increasing pH, this negative potential increased [12].

Using modified diatomite had effects on the apparent characteristics of the plant and also on the nutrients adsorbtion. In a study conducted on the effect of diatomite on the growth factors and the number of phytochemicals of beans, the plant treated with diatomite had a much better morphology in terms of shoots and roots length, number of leaves, fresh and dry weight. shoots and roots and physiological activities such as chlorophyll a and b, pigments, total soluble sugar, photosynthesis rate, total nitrogen, phosphorus, and potassium concentration were more than the treatment without diatomite [34]. Diatomite contains some water-soluble silica that can be used by plants. Diatomite is a multi-purpose material that improves the physical properties of the soil and without interfering with soil chemistry or decomposition and degradation like in other growing environments, it increases agricultural production and increases their quality. It helps to maintain and stabilize heavy metals and hydrocarbon pollutants in the environment. diatomite can increase the yield, but the condition and composition of the soil and the moisture regime play a key role in the availability of nutrients in the soil [35]. Many researchers believe that silicon is very useful for modulating biotic and abiotic stresses and even increases performance in non-stressful conditions. diatomite caused the formation of soil aggregates in soils with clay texture decreased soil density and increased soil water content. The application of diatomite improved the physical properties of the soil and, as a result, improved plant growth, including the length and number of roots and the number of leaves in strawberries. The application of silicon significantly increased the absorption of potassium and phosphorus in the aerial parts of rice [36]. The application of silica in basil plants under nickel stress increased the number of leaves, the number of lateral branches, and the height of the plant. The application of silicon causes the adjustment of nutrient deficiency, including potassium deficiency in plants [37]. The use of diatomite as a silicon source improved and increased potassium concentration in sorghum and improved plant water status. The positive effect of silicon on increasing the availability of phosphorus in the plant has been observed due to the reduction of manganese and iron absorption [38]. Silicon modifiers increase the availability of silicon, phosphorus, calcium, and magnesium for plants [52]. Using diatomite as a source of silicon increased the yield of rice, but the use of diatomite did not increase the yield of sugarcane. Such a difference for agricultural products may be due to differences in environmental conditions such as moisture regimes and geochemical conditions that can affect the solubility of Si in diatomite Silica dissolution is reduced in acidic conditions compared to alkaline conditions [35].

Diatomite treatments (2.5%, 5%), caused increasing the amount of zinc in the roots and shoots parts of the plant. The availability of zinc in the soil solution for plants is mainly dependent on pH, the presence of competing cations, and absorption sites provided by metal hydroxides, clay minerals, calcium carbonate, and organic matter. It has been reported that the application of silicon has increased the accumulation of metal in the plant. For example, the application of silicon in the soil as calcium silicate (CaSiO4) has increased the accumulation of zinc and cadmium in corn plants [39]. Silicic acid can combine with heavy metals (cadmium, lead, zinc, and mercury) in the form of soluble complex compounds and silicates of poorly soluble heavy metals. If the concentration of monosilicic acid in the soil solution increases slightly, the amount of heavy metals in the soil solution increases. The increase of this issue can increase the absorption of heavy metals, including zinc, in marigold plant. The application of silicon in the soil in the form of calcium silicate increased the accumulation of cadmium and zinc in corn plants [40].

But for other heavy metals in present research, using modified diatomite decreased the concentrations of metals in the plant tissue. Various research showed that for plants that have received enough silicon, their resistance mechanism against environmental stresses and heavy metals is very high. One of the important effects of silica on reducing the toxicity of heavy metals is reducing the absorption and transfer of metals in the plant. It has been reported by many researchers that the use of silica has increased the tolerance of many plant species to heavy metals by reducing their absorption and transport. The use of silica reduces the concentration of cadmium in different plant species such as corn [40], rice [41], wheat [42] and peanuts [43]. The effect of silica on the absorption of lead metal in banana plants, the treatment of 800 mg/kg of silica increased the pH and decreased the exchangeable lead in the soil and the concentration of lead in the roots and the aerial organs were reduced. Also silicon treatment caused to enter Pb the carbonate phase and the residue. The treatment of 100 mg/kg of silica did not affect the pH and chemical forms of lead, but both the treatment of 100 and 800 mg/kg of silica decreased the amount of lead in the banana plant. The increased tolerance of bananas to Pb toxicity was related to the stopping and stabilization of lead in the soil, the reduction of Pb transfer from the root to the shoot, and the detoxification property of silica in the plant [44]. In soil contaminated with chromium, the use of silica increased plant growth and also increased the activity of POD, SOD, and CAT enzymes in the plant. The use of silica changed the exchangeable forms of chromium into the forms bonded with organic matter. The application of silica increased the pH of the soil, which was one of the reasons for the reduction of chromium toxicity, and silica played the role of a modifier in chromium-contaminated soils [52]. The application of silicon in soil contaminated with cadmium, zinc, lead, and copper caused the immobilization of these metals in the soil and reduced their plant availability [42]. The use of silica modifiers increased the pH from 4 to 6.4 and caused a 60% decrease in the availability of metals for rice plants [45]. Reduction of Pb mobility due to the use of silica has been reported [46]. Similar results have been observed regarding cadmium and zinc in contaminated soils using silicon, which has reduced the bioavailability of metals by forming more stable fractions. Use of silicon in copper-stressed wheat caused the formation of copper complexes with organic acids and prevented the transfer of copper to shoots [47]. Diatomite as a silicon source reduced the toxicity of cadmium in wheat and reduced the available cadmium in the soil [48]. The external application of silica reduced visible stress symptoms including (low biomass and leaf chlorosis) under copper stress. Many studies have been conducted on the relationship between silica and plant tolerance to heavy metals [47, 48, 50, 52]. The main mechanisms of stress correction of heavy metals by silicon in plants include (1) entanglement or combination of metals with silicon, (2) preventing the transfer of metals from roots to buds and aerial organs, (3) division Fixation of metal ions inside the plant, (4) stimulation of antioxidant system and change of cellular structure in plants. The silicon mechanisms for modulating the stresses of heavy metals are divided into internal and external mechanisms [49]. In external mechanisms, silica modulates the toxicity of heavy metals through various methods such as reducing the absorption or activity of the metal, changing the chemical form of the metal, or increasing the Ph [45]. Under chromium stress, silica reduces chromium absorption by plants, silica increases root secretions that can chelate metals and reduce their availability [50]. The effect of nano-silica and normal silica on rice plants under Pb stress, silicon moderated lead in rice and, the lead concentration was 38.6–64.8% less than plants that did not receive silicon in their tissue [51]. Application of silicon increased the concentration of zinc, iron, and manganese in the roots of corn, wheat, and carrot plants, but decreased the concentration of copper and zinc in the aerial parts of these plants [52].

## Conclusion

This study has provided insightful findings on the use of modified diatomite for soil remediation, particularly focusing on its efficacy in heavy metal absorption and influence on nutrient dynamics within *Calendula officinalis*. As the results showed, the highest reduction in the concentrations of DTPA extractable metals was acquired when the modified diatomite was applied at the rate of 10% w/w. The results demonstrate that modified diatomite possesses significant potential in mitigating heavy metal contamination in soils, as evidenced by its high adsorption capacity for metals such as lead, cadmium, and arsenic. Furthermore, the application of modified diatomite was found to enhance the growth and development of *Calendula officinalis*, indicating an improvement in soil health and plant nutritive value. Results demonstrated, the lower amount of roots weight, shoots and roots length, and the amount of shoots and roots phosphorus, and potassium were in the control treatment. The results showed that diatomite decreased the concentration of lead, copper, nickel and chromium metals in the roots and shoot of the plant but increased the absorption of zinc metal in the roots and shoot. The highest amount of zinc absorption in roots and shoots was observed in the 2.5% (w/w) modified diatomite.

The research has underscored the dual benefits of using modified diatomite in contaminated soils: reducing the bioavailability of hazardous heavy metals and simultaneously promoting plant growth and health. This is particularly relevant for areas suffering from industrial pollution, where soil restoration is critical for sustainable agricultural practices. The study has also highlighted the environmental friendliness of this approach, as it utilizes a natural material, diatomite, innovatively, thus aligning with the principles of green remediation strategies. Future studies should focus on the long-term effects of modified diatomite application on soil health and plant growth, particularly examining the persistence of heavy metal adsorption over multiple growing seasons. Also Conducting field-scale trials will provide more comprehensive insights into the practical applicability of this remediation technique under real-world conditions, considering factors like varying soil types, climatic conditions, and large-scale logistical challenges.

## Data Availability

The datasets used and/or analysed during the current study are available from the corresponding author Maryam Samani. All data generated or analysed during this study are included in this manuscript.
